# Investigating resistance in clinical *Mycobacterium tuberculosis* complex isolates with genomic and phenotypic antimicrobial susceptibility testing: a multicentre observational study

**DOI:** 10.1016/S2666-5247(22)00116-1

**Published:** 2022-09

**Authors:** Iris Finci, Audrey Albertini, Matthias Merker, Sönke Andres, Nino Bablishvili, Ivan Barilar, Tatiana Cáceres, Valeriu Crudu, Eduardo Gotuzzo, Nchimunya Hapeela, Harald Hoffmann, Christine Hoogland, Thomas A Kohl, Katharina Kranzer, Anna Mantsoki, Florian P Maurer, Mark P Nicol, Ecaterina Noroc, Sara Plesnik, Timothy Rodwell, Morten Ruhwald, Theresa Savidge, Max Salfinger, Elizabeth Streicher, Nestani Tukvadze, Robin Warren, Widaad Zemanay, Anna Zurek, Stefan Niemann, Claudia M Denkinger

**Affiliations:** a Molecular and Experimental Mycobacteriology, Research Center Borstel, Borstel, Germany; b Evolution of the Resistome, Research Center Borstel, Borstel, Germany; c National and Supranational Reference Center for Mycobacteria, Research Center Borstel, Borstel, Germany; d FIND, Geneva, Switzerland; e German Center for Infection Research, Heidelberg, Germany; f Hamburg–Borstel–Lübeck–Riems, Germany; g National Center for Tuberculosis and Lung Diseases, Tbilisi, Georgia; h Instituto de Medicina Tropical Alexander von Humboldt, Universidad Peruana Cayetano Heredia, Lima, Perú; i Phthisiopneumology Institute Chiril Draganiuc, Chisinau, Moldova; j Division of Medical Microbiology, Institute of Infectious Disease and Molecular Medicine, University of Cape Town, Cape Town, South Africa; k SYNLAB Gauting, SYNLAB MVZ Dachau, Gauting, Germany; l Institute of Microbiology and Laboratory Medicine (IML Red), WHO Supranational TB Reference Laboratory, Gauting, Germany; m Department of Clinical Research, London School of Hygiene & Tropical Medicine, London, UK; n Biomedical Research and Training Institute, Harare, Zimbabwe; o Institute of Medical Microbiology, Virology and Hygiene, University Medical Center Hamburg-Eppendorf, Hamburg, Germany; p Division of Infection and Immunity, School of Biomedical Sciences, University of Western Australia, Perth, WA, Australia; q Division of Pulmonary, Critical Care and Sleep Medicine, University of California San Diego, La Jolla, CA, USA; r Advanced Diagnostic Laboratories, National Jewish Health, Denver, CO, USA; s Alaska State Public Health Laboratories, Anchorage, AK, USA; t College of Public Health, University of South Florida, Tampa, FL, USA; u Morsani College of Medicine, University of South Florida, Tampa, FL, USA; v DSI–NRF Centre of Excellence for Biomedical Tuberculosis Research, SAMRC Centre for Tuberculosis Research, Division of Molecular Biology and Human Genetics, Faculty of Medicine and Health Sciences, Stellenbosch University, Cape Town, South Africa; w Division of Clinical Tropical Medicine and German Centre for Infection Research, Heidelberg University Hospital, Heidelberg, Germany

## Abstract

**Background:**

Whole-genome sequencing (WGS) of *Mycobacterium tuberculosis* complex has become an important tool in diagnosis and management of drug-resistant tuberculosis. However, data correlating resistance genotype with quantitative phenotypic antimicrobial susceptibility testing (AST) are scarce.

**Methods:**

In a prospective multicentre observational study, 900 clinical *M tuberculosis* complex isolates were collected from adults with drug-resistant tuberculosis in five high-endemic tuberculosis settings around the world (Georgia, Moldova, Peru, South Africa, and Viet Nam) between Dec 5, 2014, and Dec 12, 2017. Minimum inhibitory concentrations (MICs) and resulting binary phenotypic AST results for up to nine antituberculosis drugs were determined and correlated with resistance-conferring mutations identified by WGS.

**Findings:**

Considering WHO-endorsed critical concentrations as reference, WGS had high accuracy for prediction of resistance to isoniazid (sensitivity 98·8% [95% CI 98·5–99·0]; specificity 96·6% [95% CI 95·2–97·9]), levofloxacin (sensitivity 94·8% [93·3–97·6]; specificity 97·1% [96·7–97·6]), kanamycin (sensitivity 96·1% [95·4–96·8]; specificity 95·0% [94·4–95·7]), amikacin (sensitivity 97·2% [96·4–98·1]; specificity 98·6% [98·3–98·9]), and capreomycin (sensitivity 93·1% [90·0–96·3]; specificity 98·3% [98·0–98·7]). For rifampicin, pyrazinamide, and ethambutol, the specificity of resistance prediction was suboptimal (64·0% [61·0–67·1], 83·8% [81·0–86·5], and 40·1% [37·4–42·9], respectively). Specificity for rifampicin increased to 83·9% when borderline mutations with MICs overlapping with the critical concentration were excluded. Consequently, we highlighted mutations in **M tuberculosis** complex isolates that are often falsely identified as susceptible by phenotypic AST, and we identified potential novel resistance-conferring mutations.

**Interpretation:**

The combined analysis of mutations and quantitative phenotypes shows the potential of WGS to produce a refined interpretation of resistance, which is needed for individualised therapy, and eventually could allow differential drug dosing. However, variability of MIC data for some *M tuberculosis* complex isolates carrying identical mutations also reveals limitations of our understanding of the genotype and phenotype relationships (eg, including epistasis and strain genetic background).

**Funding:**

Bill & Melinda Gates Foundation, German Centre for Infection Research, German Research Foundation, Excellence Cluster Precision Medicine of Inflammation (EXC 2167), and Leibniz ScienceCampus EvoLUNG.

## Introduction

Tuberculosis continues to be one of the leading causes of death from a single infectious agent, with an estimated 1·4 million deaths and 10 million people falling ill worldwide in 2019.^1^ Resistance to antituberculosis drugs is a major public health concern. In 2019, there were approximately half a million new cases of rifampicin-resistant tuberculosis, 78% of which had multidrug-resistant tuberculosis (defined as resistant to rifampicin and isoniazid).^1^ To successfully control tuberculosis and reduce transmission of drug-resistant *Mycobacterium tuberculosis* complex isolates, rapid detection of resistance patterns and timely initiation of appropriate treatment is crucial.^2^

Historically, phenotypic antimicrobial susceptibility testing (AST) was the reference standard; however, infrastructure, technical requirements, and the long turnaround time has affected its scale-up and impact of use.^3^ Over the past decade, AST with the use of molecular methods (eg, GeneXpert MTB/RIF [Cepheid, Sunnyvale, CA, USA]) or line-probe assays (eg, Genotype MTBDRplus and Genotype MTBDRsl [Hain Lifescience, Nehren, Germany]) has replaced phenotypic AST^4^ or has been used in parallel or sequentially, despite targeting only a small number of resistance-causing mutations. Because drug resistance of *M tuberculosis* complex isolates is caused by chromosomal variations, predominately single nucleotide polymorphisms and sometimes insertions and deletions,^5^ whole-genome sequencing (WGS) has the potential to identify all drug resistance associated mutations in a given clinical *M tuberculosis* complex isolate.^6^ WGS for resistance detection has been used extensively in research laboratories. Efforts to introduce this approach into clinical settings are ongoing.^7^ Newer WGS applications allow genome-based resistance predictions directly from acid-fast bacilli smear-positive clinical specimens rather than positive mycobacterial cultures.^8^ This process in turn speeds up time to results. Moreover, several publicly available tools can be used to interpret WGS data and identify resistance mutations^9^ to inform clinical decision making. In 2021, WHO published the first catalogue of resistance-associated mutations in clinical *M tuberculosis* complex isolates.^10^

**Research in context**

**Evidence before this study**

We searched PubMed databases for studies in all languages, published before April 1, 2021, using the search terms “*Mycobacterium tuberculosis*”, “whole genome sequencing”, “drug resistance”, “minimum inhibitory concentration” OR “phenotypic antimicrobial susceptibility testing”. The research and knowledge on using whole genome sequencing (WGS) to predict drug resistance against antituberculosis drugs have been expanding in the past decade. WGS has become an important tool for rapid diagnosis of drug resistance. Many studies provided associations between mutations in *Mycobacterium tuberculosis* complex isolates and binary drug-resistant phenotypes (resistant or susceptible). However, only a small number of publications have correlated mutations in clinical *M tuberculosis* complex isolates with quantitative measures of resistance against antituberculosis drugs using minimum inhibitory concentrations (MICs). Most of these studies included less than 100 *M tuberculosis* complex isolates, were from a single country, and investigated a small number of antituberculosis drugs. Only two studies with 176 and 72 drug-resistant *M tuberculosis* complex strains, respectively, investigated the association of mutations with MIC values for a total of 11 antituberculosis drugs (both studies: rifampicin or rifabutin, isoniazid, streptomycin, ethambutol, kanamycin, amikacin, ethionamide, moxifloxacin, and ethambutol; only in one of the studies: capreomycin, pyrazinamide, ofloxacin, cycloserine, and para-aminosalicylic acid). The larger study (n=176) had isolates from five countries: Democratic Republic of the Congo, Côte d'Ivoire, Peru, Thailand, and Switzerland, whereas the smaller study (n=72) focused only on Romania. These studies highlighted mutations associated with MICs around WHO-endorsed critical concentrations and putative wild-type strains with elevated MICs.

**Added value of this study**

By analysing many global, mostly drug-resistant, clinical *M tuberculosis* complex isolates, our study expands the current knowledge on resistance-mediating mutations and corresponding phenotypic resistance levels against selected antituberculosis drugs. We highlight mutations co-occurring with a moderate MIC increase, and which are often resulting in false phenotypic susceptibility results. In addition to the technical variability of phenotypic tests, the high variability of MICs against specific strains with identical resistance-mediating or yet undetermined mutations also shows limitations of our understanding of the genotype and phenotype correlations that need to be further investigated in well defined bacterial genetic backgrounds.

**Implications of all the available evidence**

A comprehensive database of resistance-conferring mutations and their associated resistance levels is a prerequisite if WGS is used to diagnose drug resistance in clinical practice. The results of our study will form the foundation of a knowledge base associating *M tuberculosis* complex mutations with specific MICs and will have the potential to have a substantial effect on the accuracy of genomic drug resistance prediction, and ultimately the adoption of rapid, personalised tuberculosis treatment regimens.

The sensitivity and specificity of resistance predictions, via WGS, for the first-line antituberculosis drugs rifampicin, isoniazid, ethambutol, and pyrazinamide are above 90%.^11^ Further data encompassing both phenotypic AST and WGS are needed to allow for a better link of the genotype with resistance phenotypes,^6^ particularly for second-line antituberculosis drugs.^12^ In addition, minimum inhibitory concentration (MIC) values linked to particular mutations are important to re-evaluate current clinical breakpoints, and inform individualised treatments (eg, increased drug doses to overcome so-called borderline resistance^13^, ^14^). High-quality, curated datasets derived from a globally representative *M tuberculosis* complex isolate collection are important for interrogating correlations between established cutoffs for phenotypic AST resistance detection and clinically relevant resistance.

We established a collection of multidrug-resistant and extensively drug-resistant *M tuberculosis* complex isolates from five countries (Georgia, Moldova, Peru, South Africa, and Viet Nam) with high tuberculosis prevalence and did a high-resolution WGS, phenotypic AST, and MIC analysis.^15^ WGS resistance predictions were linked with phenotypic AST and MIC data for first-line and second-line antituberculosis drugs to catalogue the effect of genomic variants on resistance levels.

## Methods

### Isolate collection

A prospective multicentre observational study to collect and store bacteriological and clinically well characterised reference materials from adult patients with drug-resistant tuberculosis was done across participating sites between Dec 5, 2014, and Dec 12, 2017. The partner sites included the Universidad Peruana Cayetano Heredia, Peru; Stellenbosch University, South Africa; University of Cape Town, South Africa; Pham Ngoc Tach Hospital, Viet Nam; the National Center for Tuberculosis and Lung Disease, Georgia; Phthisiopneumology Institute, Moldova; and the KwaZulu-Natal Research Institute for Tuberculosis and HIV, South Africa. Patients older than 18 years were included if they met any of the following criteria: (1) individuals presenting with symptoms suggestive of pulmonary tuberculosis with rifampicin resistance detected on GeneXpert MTB/RIF; (2) tuberculosis relapse cases; (3) retreatment cases after default; (4) failure of category 1 or 2 regimen (acid-fast bacilli smear positive at 5 months or later during tuberculosis treatment); or (5) multidrug-resistant tuberculosis contacts diagnosed with tuberculosis.

### Phenotypic antimicrobial susceptibility testing

*M tuberculosis* complex isolates were subcultured on Löwenstein–Jensen medium. Two laboratories used the BD BACTEC MGIT 960 (BD Diagnostic Systems Systems, Sparks, MD, USA) for phenotypic AST and to identify the MIC of 677 isolates for rifampicin, isoniazid, kanamycin, capreomycin, amikacin, moxifloxacin, levofloxacin, and pyrazinamide (appendix 1 p 18). The Institute of Microbiology and Laboratory Medicine used the Sensititre MYCOTB MIC plate (TREK Diagnostic Systems, Cleveland, OH, USA) for the identification of the MIC of 223 isolates for rifampicin, isoniazid, kanamycin, amikacin, moxifloxacin, levofloxacin, and ethambutol (appendix 1 p 18).

### WGS and molecular drug resistance prediction

WGS was done with Illumina (San Diego, CA, USA) technology according to the manufacturer's instructions. Raw read data (Acc.No PRJEB48275) were mapped to the *M tuberculosis* H37Rv genome (GenBank accession number NC_000962.3) with MTBseq^16^ and aimed for at least 50 times the average genome-wide coverage. Detailed analysis parameters are provided in appendix 1 (pp 2–3). We report on the predictions of resistance against antituberculosis drugs and associated resistance genes: rifampicin (*rpoB*), isoniazid (*fabG1, fabG1* promotor*, inhA, ndh, katG, mshA, ahpC,* and *ahpC* promotor), levofloxacin and moxifloxacin (*gyrA* and *gyrB*), kanamycin (*rrs* and *eis* promotor), amikacin (*rrs*), capreomycin (*rrs* and *tlyA*), ethambutol (*embCAB* operon), and pyrazinamide (*pncA, pncA* promotor, and *rpsA*). Genotypic resistance was inferred on the basis of a curated mutation catalogue used at the Supranational Reference Laboratory, Research Center Borstel, Germany, based on information available on May 10, 2020.^17^

### Data analysis

Data analyses were done with the R software version 4.0.3. Full binary phenotypic resistance profiles of each isolate were inferred from the MIC data on the basis of the previous WHO classification.^18^ Not all isolates had MIC results for all nine antimicrobials; for isolates without MIC results, information on antimicrobial resistance and susceptibility was added from available binary phenotypic AST results. For levofloxacin, 17 isolates were tested at the previously endorsed critical concentration^19^ of 1·5 mg/L and MIC dilutions did not include the new critical concentration of 1·0 mg/L. These isolates were excluded from the sensitivity and specificity analysis as their phenotypic AST result was not interpretable. To calculate sensitivity and specificity, WGS-based resistance prediction was compared with phenotypic AST results with the WHO-endorsed critical concentrations^19^ from 2018, where critical concentration for rifampicin was 1·0 mg/L (appendix 1 p 18). For the graphical representations of the rifampicin and isoniazid MIC distributions (appendix 1 pp 6–7), we excluded 22 rifampicin isolates and 12 isoniazid isolates due to a truncated lower MIC border. Phylogenetic relationships of all analysed *M tuberculosis* complex isolates were inferred from hierarchical clustering on the basis of a distance matrix of 33 605 single nucleotide polymorphisms. Two isolates with *rpoB* Ser450Leu mutation and a drug-susceptible phenotype for rifampicin and four isolates with *katG* Ser315Thr mutation and a drug-susceptible phenotype for isoniazid were removed from the analysis as they most likely represent labelling error rather than methodical error.

### Ethical approval

Ethical approvals for the study were obtained from the Viet Nam Committee of the Ministry of Health, Phthisiopneumology Institute Chiril Draganiuc, Moldova; Faculty of Medicine and Health Sciences Human Research, Stellenbosch University, South Africa; Human Research Ethics Committee, Faculty of Health Sciences Human Research, University of Cape Town, South Africa; Biomedical Research, University of KwaZulu-Natal, South Africa; Peruvian National Center for Tuberculosis and Lung Diseases; and the Universidad Peruana Cayetano Heredia, Peru. The study was undertaken in accordance with the principles of the Helsinki Declaration.^20^ Written informed consent was obtained from patients who agreed to participate.

### Role of the funding source

The funders of the study had no role in the study protocol, data analysis, data collection, data interpretation, or writing of the manuscript.

## Results

WGS and phenotypic AST testing (including MIC) for at least one of nine antituberculosis drugs was successfully done for 900 *M tuberculosis* complex isolates (table 1, figure). The *M tuberculosis* complex isolates originated from different geographical regions: 35·1% from eastern Europe (Georgia and Moldova), 31·3% from southern Africa, 21·9% from southeast Asia (Viet Nam), and 11·7% from South America (Peru). On the basis of the phenotypic AST results, the resistance profiles of the 900 *M tuberculosis* complex isolates were classified according to the WHO classification:^18^ 66 (7·3%) as drug-susceptible, 77 (8·5%) as monoresistant or polyresistant, 613 (68·1%) as multidrug resistant, and 144 (16·0%) as extensively drug resistant.^21^ Phylogenetic analysis based on single nucleotide polymorphisms identified the following lineages (L) and sublineages:^22^ L2 (57·4%), L4.2 (13·8%), L4.3 (10·9%), L4.1 (10·1%), L4.8 (3·6%), L4.4 (1·8%), L1 (1·1%), and other (1·3%). Most extensively drug-resistant isolates were L2 (83·3%) and the majority of multidrug-resistant isolates belonged to L2 (57·1%), L4.2 (18·1%), and L4.3 (11·7%). Most extensively drug-resistant isolates were from South Africa (82·6%), whereas multidrug-resistant isolates originated from Moldova (32·3%), Viet Nam (27·4%), South Africa (14·9%), Peru (12·9%), and Georgia (12·6%; figure).

**Table 1:** Characteristics of the analysed *Mycobacterium tuberculosis* complex isolates

		**Isolates**
**Drugs with MIC results**		
Isoniazid		898/900 (99·8%)
Rifampicin		898/900 (99·8%)
Kanamycin		895/900 (99·4%)
Amikacin		895/900 (99·4%)
Capreomycin		672/900 (74·7%)
Moxifloxacin		897/900 (99·7%)
Levofloxacin		742/900 (82·4%)
Ethambutol*		223/900 (24·8%)
Pyrazinamide*		213/900 (23·7%)
**Resistance profile (based on phenotypic data)**		
Drug susceptible		66/900 (7·3%)
Monoresistance or polyresistance		55/900 (6·1%)
Polyresistant second-line drugs†		22/900 (2·4%)
MDR		613/900 (68·1%)
XDR		144/900 (16·0%)
**Lineage**		
Lineage one (East African—Indian)		10/900 (1·1%)
Lineage two (Beijing)		517/900 (57·4%)
Lineage four (Euro—American)		373/900 (41·4%)
**Country (based on phenotypic data)**		
Moldova		219/900 (24·3%)
	MDR	198/613 (32·3%)
	XDR	9/144 (6·3%)
Georgia		97/900 (10·8%)
	MDR	77/613 (12·6%)
	XDR	12/144 (8·3%)
Peru		105/900 (11·7%)
	MDR	79/613 (12·9%)
	XDR	2/144 (1·4%)
South Africa		282/900 (31·3%)
	MDR	91/613 (14·9%)
	XDR	119/144 (82·6%)
Vietnam		197/900 (21·9%)
	MDR	168/613 (27·4%)
	XDR	2/144 (1·4%)

MDR=multidrug resistant. XDR=extensively drug resistant. MIC=minimum inhibitory concentration.

* Additional samples without MIC results and only phenotypic antimicrobial susceptibility testing results (resistant or susceptible) were available.

† Includes isolates with resistance to either isoniazid or rifampicin, and resistance to at least one fluoroquinolone, second-line injectable drug, or to both.

**Figure:**
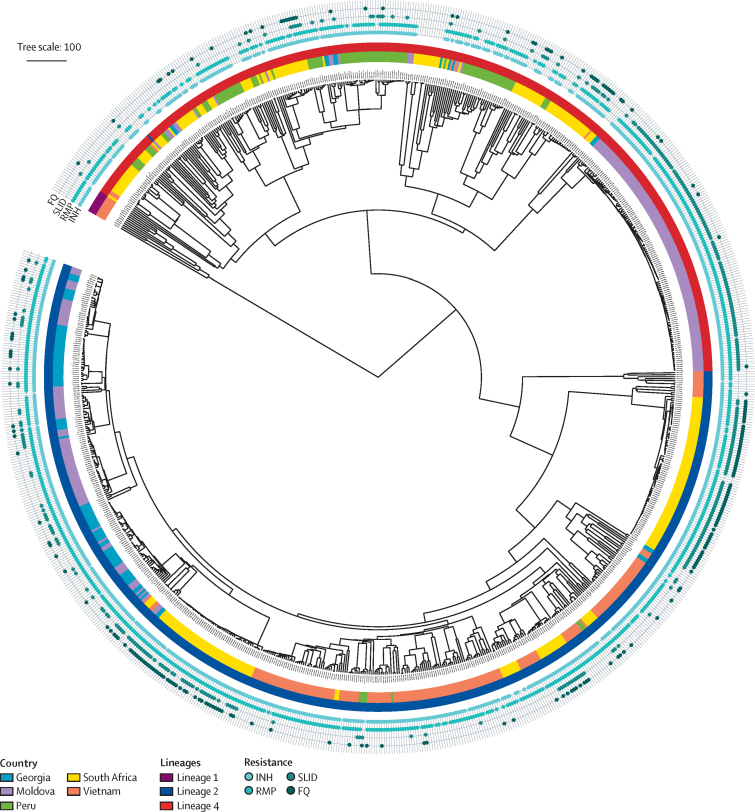
Phylogenetic relationship, origin, and individual drug resistances of 900 *Mycobacterium tuberculosis* complex isolates The inner circle represents lineages. The circle in the middle represents origin countries of the samples. The outer circle consists of four lines with filled circles representing resistance to the following antibiotics: INH, RMP, SLID (kanamycin, capreomycin, or amikacin), and FQ (moxifloxacin or levofloxacin); order from inside towards outside. For both SLID and FQ, strains with resistance to at least one antibiotic were considered as resistant. INH=isoniazid. RMP=rifampicin. SLID=second-line injectable drugs. FQ=fluoroquinolones.

To visualise the association between particular mutations and the resistance level, we generated mutation and MIC plots for all antituberculosis drugs on the basis of the mutations defined by WGS (appendix 1 pp 5–14). After excluding purely phylogenetic polymorphisms^23^ and synonymous mutations, we observed 3197 mutations with potential association with resistance (appendix 2).^17^

A visual inspection of the mutation and MIC plots showed a high variability in resistance levels associated with specific mutations for the relevant drug (appendix 1 pp 6–14). For each drug tested, there were well documented resistance-conferring mutations known to confer high levels of resistance (eg, for rifampicin, the MIC was ≥16 mg/L for mutation *rpoB* Ser450Leu; appendix 1 p 6), and a large number of mutations associated with lower MIC levels. Several of these mutations result in an MIC near to the critical concentration (eg, *rpoB* Asp435Val and His445Leu; table 2), and some mutations with MICs just below the critical concentration (eg, *rpoB* Leu430Pro or Asp435Tyr; table 3). If these mutations occurred together with a second *rpoB* mutation, the MIC level increased to above critical concentration values, inferring a possible stepwise acquisition of high-level resistance (appendix 1 p 15). A similar observation was made for isoniazid: isolates with the mutation –15 C→T in the *fabG1* promoter region had only a moderate resistance level (<1 mg/L), whereas isolates with mutations in *katG* Ser315Thr had high-level isoniazid resistance (2–4 mg/L). In isolates with both mutations present, MICs were further elevated, suggesting additive resistance (predominantly ≥10 mg/L via the mycobacterial growth indicator tubes and ≥4 mg/L via MycoTB; appendix 1 p 7).

**Table 2:** Gene mutations associated with MICs overlapping the critical concentration of each antituberculosis drug^21^

		**Mutation**	**Isolates**	**Susceptible isolates**	**Observed MIC range (mg/L)**
Rifampicin					
	*rpoB*	Asp435Val	68	9	0·5 to ≥20
	*rpoB*	His445Leu	6	4	1·0 to 4·0
Kanamycin					
	*eis*	−10 G→A	18	4	0·63 to 12·5
	*eis*	−12 C→T	121	13	1·25 to 12·5
	*eis*	−37 G→T	11	2	2·5 to 12·5
	*eis*	−14 C→T	5	1	1·25 to ≥25
Amikacin					
	*eis*	−14 C→T	12	8	0·5 to 4·0
Capreomycin					
	*rrs*	1401 A→G	25	6	1·25 to ≥12·5
Moxifloxacin					
	*gyrA*	Ala90Val	50	9	0·25 to 4·0
Levofloxacin					
	*gyrA*	Ala90Val	27	3	0·75 to 8·0
Ethambutol					
	*embB*	Met306Ile	79	54	1·0 to 16·0
	*embB*	Met306Val	65	16	2·0 to 16·0
	*embB*	Gln497Arg	7*	5	4·0 to 8·0
Pyrazinamide					
	*pncA*	Leu19Pro	2	1	100 to 200
	*pncA*	Asp63Ala	3	1	100 to 200
	*pncA*	Val7Ala	2*	1	50 to 200
	*pncA*	Val180Ala	4*	2	100 to >400

Critical concentrations for these drugs are: rifampicin (1·0 mg/L), isoniazid (0·1 mg/L), kanamycin (2·5 mg/L), amikacin (1·0 mg/L), capreomycin (2·5 mg/L), moxifloxacin (0·25 mg/L), levofloxacin (1·0 mg/L), ethambutol (4·0 mg/L), and pyrazinamide (100 mg/L). MIC=minimum inhibitory concentration.

* Clonal populations (difference of less than 12 single nucleotide polymorphisms between isolates).

**Table 3:** Isolates with resistant genotypic AST classification and MIC values exclusively below or equal to the critical concentration of each antituberculosis drug^21^ (false positive predictions)

	**Mutation**	**Isolates**	**Isolates in a cluster with ≤12 differences in single nucleotide polymorphisms**	**MIC, mg/L (number of isolates, method)**
**Rifampicin**				
*rpoB*	Leu452Pro	6	0	0·25 (1, MycoTB), 0·5 (5, MGIT)
*rpoB*	Leu430Pro	8	2	0·13 (4), 0·25 (1), 1·0 (3), all MGIT
*rpoB*	Asp435Tyr	4	2	0·13 (1, MycoTB), 0·5 (2, MGIT), 1·0 (1, MGIT)
*rpoB*	His445Asn	3	2	0·13 (1), 0·5 (2), all MGIT
**Moxifloxacin**				
*gyrB*	Ala504Val	2	0	≤0·06 (1), 0·25 (1)
**Levofloxacin**				
*gyrB*	Ala504Val	2	0	≤0·19 (1), 0·75 (1), all MGIT
*gyrB*	Thr500Asn	1	..	0·75 (MGIT)
*gyrA, gyrB*	Val457Leu (*gyrB*) + Ser91Pro (*gyrA*)	2	0	0·5 (1), 1·0 (1), all MycoTB
*gyrB*	Asp461His + Val457Leu	1	..	1·0 (MycoTB)
**Ethambutol (all MycoTB)**				
*embB*	Gly406Asp	4	4	2–4
*embB*	Gly406Ala	1	..	2
*embB*	Gly406Ser	1	..	2
*embB*	Gly406Cys	1	..	2
**Pyrazinamide (all MGIT)**				
*pncA*	Ala28Thr	1	..	100
*pncA*	Thr47Ala	1	..	50
*pncA*	Asp63Gly	2	2	100
*pncA*	Asp136Ala	1	..	100
*pncA*	Asp136Gly	1	..	100
*pncA, rpsA*	Ala412Val (*rpsA*) + Val155Ala (*pncA*)	1	..	50
*pncA, rpsA*	Asp54Gly (*rpsA*) + Pro69Ser (*pncA*)	1	..	50

Mutations with MICs overlapping the critical concentration are not included. Critical concentrations for these drugs are: rifampicin (1·0 mg/L), isoniazid (0·1 mg/L), kanamycin (2·5 mg/L), amikacin (1·0 mg/L), capreomycin (2·5 mg/L), moxifloxacin (0·25 mg/L), levofloxacin (1·0 mg/L), ethambutol (4·0 mg/L), and pyrazinamide (100 mg/L). AST=antimicrobial susceptibility testing. MIC=minimum inhibitory concentration. MGIT=mycobacteria growth indicator tube. MycoTB=Sensititre MYCOTB MIC plate.

The data also showed the variability of resistance levels associated with mutations mediating resistance against a fluoroquinolone (eg, *gyrA* Glu501Asp; appendix 1 p 15), or mutations in *rrs* and the *eis* promoter regions, mediating resistance against second-line injectable drugs (appendix 1 pp 8–10). Particularly for kanamycin, the mutation *rrs* 1401 A→G caused high-level resistance, mainly with MIC 25 mg/L or higher, whereas the mutation –12 C→T in the *eis* promoter resulted in a moderate kanamycin MIC increase to 5 mg/L in most isolates (appendix 1 p 8). Likewise, for amikacin, *rrs* 1401 A→G caused high-level resistance (MIC ≥16 mg/L), whereas only *eis* –14 C→T appeared to have had only a mild effect on amikacin MICs (appendix 1 p 9). For both moxifloxacin and levofloxacin, the MIC was elevated when Asp94Asn, Asp94Tyr, and Gly88Cys mutations in *gyrA* were present compared with the wild types (appendix 1 pp 11–12). Moreover, mutations in *gyrB* Glu501Asp, Thr500Ala, and Thr500Asn were associated with resistance to moxifloxacin, but not with resistance to levofloxacin at the current critical concentrations. Mutations associated with lower MIC levels also had an effect on the specificity of genotypic AST resistance predictions made from the WGS mutation data for all isolates.

The sensitivity of WGS for predicting resistance (ie, the proportion of phenotypic drug-resistant isolates for which genotypic AST yielded a resistance marker) was high (table 4). For core first-line antituberculosis drugs, the genotypic AST sensitivity was 98·8% for isoniazid and 99·5% for rifampicin. Sensitivity for second-line injectable drugs was slightly lower: 96·1% for kanamycin, 97·2% for amikacin, and 93·1% for capreomycin. Sensitivity of levofloxacin genotypic AST prediction for resistance was 94·8%, whereas sensitivity for moxifloxacin resistance was slightly lower at 88·9%. Lastly, ethambutol resistance prediction achieved 100% sensitivity, whereas pyrazinamide resistance prediction had the lowest sensitivity at 87·9%.

**Table 4:** Sensitivity and specificity of antituberculosis drug resistance predictions based on whole-genome sequence compared with the MICs of phenotypic AST of each antituberculosis drug

	**Susceptible isolates (phenotypic AST)**	**Resistant isolates (phenotypic AST)**	**Isolates without genotypic AST resistance marker**	**Isolates with genotypic AST resistance marker**	**Sensitivity (95% CI)**	**Specificity (95% CI)**
Isoniazid*	88	808	95	801	98·8% (98·5–99·0)	96·6% (95·2–97·9)
Rifampicin*	114	780	77	817	99·5% (99·3–99·7)	64·0% (61·0–67·1)
Kanamycin	564	331	549	346	96·1% (95·4–96·8)	95·0% (94·4–95·7)
Amikacin	714	181	709	186	97·2% (96·4–98·1)	98·6% (98·3–98·9)
Capreomycin	600	29	592	37	93·1% (90·0–96·3)	98·3% (98·0–98·7)
Moxifloxacin	681	216	686	211	88·9% (87·4–90·3)	97·2% (96·8–97·6)
Levofloxacin†	628	96	615	109	94·8% (93·3–97·6)	97·1% (96·7–97·6)
Ethambutol	142	81	166	57	100% (100)	40·1% (37·4–42·9)
Pyrazinamide	80	132	85	127	87·9% (86·0– 89·8)	83·8% (81·0–86·5)

MIC=minimum inhibitory concentration. AST=antimicrobial susceptibility testing.

* Due to potential laboratory error, four samples with Ser450Leu mutation in *rpoB* (rifampicin) and two samples with Ser315Thr in *katG* (isoniazid) were excluded from the analysis.

† 17 samples were tested at MIC 1·5 mg/L (previous critical concentration) and were excluded from the analysis.

Specificity of WGS for predicting resistance (ie, the proportion of phenotypic susceptible isolates for which genotypic AST yielded no resistance markers) was high for isoniazid (96·6%), kanamycin (95·0%), amikacin (98·6%), capreomycin (98·3%), moxifloxacin (97·2%), and levofloxacin (97·1%). However, the specificity of resistance prediction for pyrazinamide was only 83·8%, and was even lower for rifampicin (64·0%) and ethambutol (40·1%). After excluding individual borderline mutations that had elevated MICs (compared with wild-type isolates), but which often tested susceptible at the WHO-endorsed critical concentrations^19^ of 1·0 mg/L (Leu430Pro, Asp435Tyr, His445Asn, or Leu452Pro), specificity for rifampicin resistance detection increased to 83·9% (95% CI 81·3–86·6).

We then used our high-resolution mutation and MIC plots to identify the effect of particular mutations on the MICs of all drugs investigated, and to define the specific mutations leading to MICs which were higher, but less than the critical concentration. These mutations were also considered to potentially lead to false susceptible phenotypic AST results using the current critical concentrations as reference for resistance detection. The respective mutation and MIC plots are shown in appendix 1 (pp 6–14, 16).

Isolates with MICs above the critical concentrations, for which WGS did not identify any resistance-conferring mutation based on the reference mutation catalogue, were considered false susceptible in the genotypic AST (table 5). Individual mutations (not part of the resistance mutation database) such as Ser450Met in *rpoB*, Asp94Leu in *gyrA*, and Asn499Thr and Thr500Ala in *gyrB* occurred in canonical codon positions with uncommon or yet unknown amino acid changes and most likely represent novel resistance-mediating mutations. Other potential candidates need to be further explored (table 3). Some of the mutations previously not associated with resistance were associated with an MIC around the critical concentrations. These mutations were linked to moxifloxacin resistance, such as Ala288Asp in *gyrA*, and Pro94Leu, Arg446Cys, Ser447Phe, Ser447Tyr, and Asp461Asn in *gyrB*, and to pyrazinamide resistance, such as *pncA* Ala102Val and *rpsA* Met432Thr (appendix 1 p 16). Several isolates with genotypic AST wild types were identified to have MICs above the critical concentration value for different drugs. In some of these isolates, resistance mutations present below the set thresholds were detected (appendix 1 pp 16–17), suggesting a mixed *M tuberculosis* complex population. In others, low frequency mutations were not detected.

**Table 5:** Isolates with susceptible genotypic AST classification and MIC values exclusively above the critical concentration of each antituberculosis drug (false negative predictions)

	**Mutation (tier 1)**	**Mutation (tier 2)**	**Isolates**	**Isolates in a cluster with ≤12 differences in single nucleotide polymorphisms**	**Critical concentrations, mg/L**	**MIC, mg/L (number of isolates, method)**
Rifampicin	*rpoB* His194Tyr	*rpoC* Ala172Val, Arg173Arg, Pro601Leu, *Rv2752c* Ala520Ala	1	..	1	>20 (MGIT)
Rifampicin	*rpoB* Ser450Met*†	*rpoC* Gly594Glu	1	..	1	>20 (MGIT)
Rifampicin	*rpoB* WT	All WT	2	..	1	≥20 (MGIT)
Isoniazid	*ahpC* −142 G→A	All WT	1	..	0·1	3 (MGIT)
Isoniazid	*katG* Asp419His	All WT	1	..	0·1	0·4 (MGIT)
Isoniazid	*ahpC* −52 C→T *+ katG* Arg187Trp	All WT	1	..	0·1	≥10 (MGIT)
Isoniazid	*katG* Ser383Ala + Tyr337Ser	All WT	1	..	0·1	0·4 (MGIT)
Isoniazid	*katG, ahpC, inhA, fabC1, ndh, mshA,* all WT	All WT	6	..	0·1	0·25 (1, MycoTB), 1 (1, MGIT), 3 (2, MGIT), >4 (2, MGIT)
Kanamycin	*rrs*1443 C→T	No tier 2 gene defined	1	..	2·5	≥40 (MycoTB)
Kanamycin	*rrs, eis, whiB7,* all WT	No tier 2 gene defined	11	4 (2 clusters of 2 isolates)	2·5	5 (6, MGIT), 12·5 (3, MGIT), ≥40 (2, MycoTB)
Amikacin	*rrs*1443 C→T	All WT	1	..	1	≥16 (MycoTB)
Amikacin	*rrs, eis,* all WT	All WT	1	..	1	4 (1, MGIT)
Amikacin	*rrs, eis,* all WT	*ccsA9* Ile245Met + *aftB* Asp397Gly, *ccsA* Val27Ile + *aftB* Asp397Gly	11	..	1	≥40 (1, MGIT), ≥16 (1, MycoTB),
Capreomycin	*rrs, tlyA,* all WT	All WT	1	..	2·5	5 (MGIT)
Moxifloxacin	*gyrA* Asp94Leu†	No tier 2 gene defined	1	..	0·25	4 (MycoTB)
Moxifloxacin	*gyrB* Asn499Thr†	No tier 2 gene defined	1	..	0·25	0·5 (MGIT)
Moxifloxacin	*gyrB* Thr500Ala†	No tier 2 gene defined	1	..	0·25	0·5 (MGIT)
Moxifloxacin	*gyrA, gyrB, all WT*	No tier 2 gene defined	9	3	0·25	0·5 (3, MGIT), 1 (2, MycoTB), 2 (2, MycoTB), 4 (1, MycoTB), ≥8 (1, MycoTB)
Levofloxacin	*gyrA* Asp94Leu†	No tier 2 gene defined	1	..	1	4 (MycoTB)
Levofloxacin	*gyrA, gyrB, all WT*	No tier 2 gene defined	3	..	1	2 (2, MycoTB), 8 (1, MycoTB)
Pyrazinamide	*pncA, rpsA, panD, clpC1, all WT*	All WT	11	2	100	400 (3, MGIT), >400 (8, MGIT)
Pyrazinamide	*pncA, rpsA, panD, clpC1, all WT*	*Rv3236c* Tyr200Tyr	1	..	100	>400 (1, MGIT)

Mutations with MICs overlapping the critical concentration are not included. AST=antimicrobial susceptibility testing. MIC=minimum inhibitory concentration. MGIT=mycobacterium growth indicator tube. WT=wildtype. MycoTB=Sensititre MYCOTB MIC plate.

* A comparison to the latest WHO mutation catalogue^10^ supported the relevance of *rpoB* Ser450Met to be associated with resistance against rifampicin.

† These mutations occur in canonical codon positions highly associated with drug resistance but individual amino acid changes are rarely observed in clinical isolates or are not yet reported.

The specificity of genotypic AST for prediction of rifampicin resistance was affected by a set of borderline mutations that lead to a raised MIC but below the critical concentrations in liquid medium, while testing resistant in solid medium.^24^, ^25^ In our study, this effect was clearly seen for several *rpoB* mutations, significantly effecting the specificity of genotypic AST for rifampicin resistance prediction (table 4, appendix 1 p 15). For example, presence of Leu430Pro, Asp435Tyr, His445Asn, or Leu452Pro mutations in the *rpoB* gene without any other mutation in *rpoB* did not increase the MIC above the critical concentrations (appendix 1 pp 6, 15). Furthermore, isolates with *rpoB* Asp435Val and His445Leu mutations showed MIC distributions around the critical concentrations—ie, sometimes including susceptible and resistant MIC value classifications (table 5, appendix 1 p 6).

For the injectable drugs kanamycin and amikacin, the MIC distribution of isolates harbouring mutations in the *eis* promoter overlapped with the critical concentrations, lowering the specificity of the resistance prediction. These mutations were: –10 G→A, –12 C→T, –14 C→T, and –37 G→T for kanamycin and –14 C→T for amikacin. For capreomycin, isolates with 1401 A→G mutation in *rrs* had MIC distribution overlapping the critical concentrations. For both moxifloxacin and levofloxacin, isolates with Ala90Val in *gyrA* had varying MICs (table 5).

Ethambutol genotypic AST had the lowest specificity for resistance prediction of all analysed drugs, largely due to two mutations at the same amino acid position, Met306Ile and Met306Val in the *embB* gene. Here, the MIC distribution overlapped the critical concentrations, resulting in some isolates phenotypically susceptible but predicted to be resistant (table 5, appendix 1 p 13). Lastly, several mutations associated with either variable MIC or false positive pyrazinamide resistance prediction were detected.

## Discussion

In this study, we analysed 900 mostly rifampicin-resistant *M tuberculosis* complex isolates from five countries across four continents) and did WGS and MIC testing for nine drugs. Our data suggest that with the current knowledge available, high accuracy can be achieved using genotypic AST for first-line and most second-line antituberculosis drugs. We provide refined knowledge of resistance mutation linked to quantitative MIC results which shows how individual mutations and combinations of mutations affect the resistance level. Such data are urgently needed to pave the way to guide individualised tuberculosis treatment, and re-evaluate current critical concentrations, especially in light of borderline resistance mutations. Importantly, the data drive further research to characterise newly identified mutations and alternative resistance mechanisms, and can inform the prioritisation of mutations to be included in future molecular AST assays.

Our results inform a more nuanced understanding of borderline mutations, associated with a moderate MIC increase or MICs below currently established critical concentrations in liquid medium. For rifampicin, the presence of borderline mutations (eg, *rpoB* Leu430Pro, Asp435Tyr, His445Asn, and Leu452Pro) has been clearly associated with worse outcomes,^24^, ^26^ and the effect of borderine mutations on treatment outcomes is also discussed for quinolones, second-line injectable drugs, and ethambutol.^27^, ^28^

Furthermore, we show that the specificity of ethambutol resistance prediction was greatly reduced by mutations in the *embB* gene, Met306Val and Met306Ile, for which MIC distributions overlapped with the critical concentrations. With regard to the low specificity to predict rifampicin resistance in our study, WHO acknowledged that the previously recommended critical concentrations of 1 mg/L in the mycobacterial growth indicator tubes was too high and resulted in mutant isolates being falsely classified as susceptible.^29^ This breakpoint artifact has been also observed in a study in China which used quantitative Sensititre MYCOTB plates.^30^ Also, different mutations in *gyrB* at amino acid positions 499–501 (the quinolone binding pocket) exhibit variable effects on the resistance level to different fluoroquinolones, in line with a previous study.^31^ We also confirmed results of other studies suggesting that the *gyrB* Glu501Asp mutation confers resistance to moxifloxacin but not to levofloxacin at the currently endorsed critical concentrations.^31^ We expand the number of candidate mutations that have the same effect: *gyrB* Thr500Ala or *gyrB* Thr500Asn. Further studies are needed to define the prevalence of these mutations, and most importantly the presence of specific mutations must be correlated with clinical outcomes. Known borderline mutations are indeed emerging in phylogenetically unrelated multidrug-resistant isolates from different geographical regions, which is a clear signal of positive selection and the functional effect.^32^, ^33^, ^34^ Consequently, in the absence of clear evidence that endorsed drug concentrations are effective against strains with borderline mutations, borderline mutations need to be considered as potential resistance determinants. In some cases, decreased drug susceptibility might be overcome by increasing the drug dosage and exposure, similar to what is already practised in cases of isoniazid when *inhA* mutation is present.^35^

We also detected novel mutations (eg, *rpoB* Ser450Met) potentially conferring resistance that could be added to existing resistance databases. However, each of these mutations was found in a single isolate, suggesting they might be of low clinical importance (but could be selected for, with drug pressure, in the future) and the results need to be investigated further (table 5, appendix 1 p 16).^36^

A limitation of this analysis is the use of an existing resistance catalogue and defined target genes, instead of employing the whole genome as a knowledge-generating base. However, although a wider association study was beyond the scope of this study, the data generated here can be used to rapidly complement ongoing and previous genome-wide association studies. The need for unbiased approaches to close existing gaps in our knowledge of the genotype and phenotype relationship was shown by *M tuberculosis* complex isolates with elevated MICs but lacking a mutation from our resistance catalogue. In this case, new resistance mechanisms, such as epistasis, or an effect of the strain genetic background might explain elevated MICs. Moreover, the study used two different methods to estimate MICs, and sensitivity and specificity between the two methods differed. Isolates used in this study are biased towards rifampicin and isoniazid resistance, and lineages two and four isolates, reflecting the *M tuberculosis* complex population structure in our study settings and the general diagnostic focus in high tuberculosis incidence settings to apply AST assays predominantly on drug-resistant clinical specimens. In future research, more equal representations of all lineages should be attempted, especially to elucidate the potential effect of the genetic background on the MIC level of strains with identical resistance-conferring mutations. Also, the presence of mixed infections below the detection threshold could result in false negative genotypic AST results. Currently, there are advances in detecting mixed *M tuberculosis* complex populations from clinical isolates; however, such analysis was also outside of the scope of this study. Particularly for rare mutations, laboratory errors can have a significant effect, and differences between MIC methodologies in the different laboratories could be responsible for some of the variability observed in the MIC data of strains with identical resistance-mediating mutations.

Nevertheless, our data support the substantial progress made with WGS for resistance prediction in clinical *M tuberculosis* complex isolates. We confirm high accuracy of genotypic AST for the drugs investigated, in line with a study of the Comprehensive Resistance Prediction for Tuberculosis consortium making similar observations for first-line drugs only.^11^ Using data from global sources, WHO is also extending the database to correlate genotypic and phenotypic information, and has generated an amended list of resistance-conferring mutations.^10^

In conclusion, our study compiled a unique dataset to address the pressing question of whether genotypic AST is a valuable tool for individualised tuberculosis therapy. Our data clearly show the potential of using genotypic AST, not only for high-resolution susceptibility and resistance profiling of clinical *M tuberculosis* complex isolates, but also for highlighting the weakness of binary phenotypic AST results. Considering both, the resistance genotype and phenotypic MIC values will help to resolve uncertainties around the current critical concentrations for some drugs and will be crucial in the future for individual drug dosing and precision medicine for patients with drug-resistant tuberculosis. Our results are informing larger databases, which will enable the transition from binary phenotypic AST towards WGS-based resistance prediction, and will support rapid, personalised treatment decisions.

## Data sharing

All data used in this study and European Nucleotide Archive accession numbers for the raw sequencing data for all next-generation sequencing datasets are provided in appendix 2.

## Declaration of interests

MM and SN report grants from the German Center for Infection Research, Excellenz Cluster Precision Medicine in Chronic Inflammation, and Leibniz Science Campus Evolutionary Medicine of the LUNG (EvoLUNG). TR reports personal fees from FIND, grants from the US National Institute of Allergy and Infectious Diseases, and is a board member for Verus Diagnostics; and has a provisional patent (#63/048.989) and a pending patent (#14840432.0) for tuberculosis diagnostics. All other authors declare no competing interests.
