# Supplementary material for: Investigating resistance in clinical Mycobacterium tuberculosis complex isolates with genomic and phenotypic antimicrobial susceptibility testing: a multicentre observational study
Source: Lancet Microbe. 2022 Sep;3(9):e672–82. doi: 10.1016/S2666-5247(22)00116-1 (PMC9436784; doi:10.1016/S2666-5247(22)00116-1)
Supplement: Supplementary appendix 1 [file mmc1.pdf]

THE LANCET
Microbe

Supplementary appendix 1

This appendix formed part of the original submission and has been peer reviewed. We post it as supplied by the authors.

Supplement to: Finci I, Albertini A, Merker M, et al. Investigating resistance in clinical *Mycobacterium tuberculosis* complex isolates with genomic and phenotypic antimicrobial susceptibility testing: a multicentre observational study. *Lancet Microbe* 2022; published online July 27. https://doi.org/10.1016/S2666-5247(22)00116-1.

Investigating resistance in clinical Mycobacterium tuberculosis complex isolates with genomic and phenotypic antimicrobial susceptibility testing: a multicentre observational study

Supplementary methods

Methods **2**

Specimen collection, storage and shipment *2*

Phenotypic antimicrobial susceptibility testing *2*

Whole genome sequencing and molecular drug resistance prediction *2*

Data analysis *3*

Table S1. Genes analyzed for presence of antibiotic resistance mutations 5
Table S4. Sensitivity and specificity of WGS-based antibiotic resistance prediction compared to phenotypic DST – MIC performed on MGIT and MycoTB 5
Figure S1. Rifampicin drug resistance mutations with corresponding MIC tested with MGIT and MycoTB 6
Figure S2. Isoniazid drug resistance mutations with corresponding MIC tested with MGIT and MycoTB 7
Figure S3. Kanamycin drug resistance mutations with corresponding MIC tested with MGIT and MycoTB 8
Figure S4. Amikacin drug resistance mutations with corresponding MIC tested with MGIT and MycoTB 9
Figure S5. Capreomycin drug resistance mutations with corresponding MIC tested with MGIT 10
Figure S6. Moxifloxacin drug resistance mutations with corresponding MIC tested with MGIT and MycoTB 11
Figure S7. Levofloxacin drug resistance mutations with corresponding MIC tested with MGIT and MycoTB 12
Figure S8. Ethambutol drug resistance mutations with corresponding MIC tested with MycoTB 13
Figure S9. Pyrazinamide drug resistance mutations with corresponding MIC tested with MGIT 14
Figure S10. Mutation E501D in gyrB causing different MIC in moxifloxacin and levofloxacin 15
Figure S11. MIC distribution for strains with mutations L452P and L430P in rpoB alone or with other mutations in rpoB 15
Table S5. MIC close to the critical concentration 16
Table S6. Examination of low frequency or low coverage mutations in strains with antibiotic resistance predicted based on pDST where gDST characterization predicted WT strains 16
Table S7. Concentrations used for testing performed in BD MGIT 960 18
Table S8. Concentrations used for testing performed in Sensititre MycoTB plate 18

References **19**

Methods

Specimen collection, storage and shipment

Up to three sputum specimens (and at least 2 with ≥ 3ml) collected at baseline were processed for each participant. Mtbc isolates from positive Mycobacterium Growth Indicator Tubes (MGIT, BD, USA) or Löwenstein-Jensen (LJ) slant cultures were either stored and shipped directly, or shipped after transfer into glycerol stocks (1 ml) from the enrolment centers to one of three reference laboratories, i.e. National Reference Center for Mycobacteria in Germany, National Jewish Health in the US, or the Institute of Microbiology and Laboratory Medicine (IML) in Germany, for pAST and gAST (see below).

Phenotypic antimicrobial susceptibility testing

Mtbc isolates were sub-cultivated on LJ medium. Two laboratories used MGIT960 to perform pAST and to determine the MIC of 677 isolates for rifampicin, isoniazid, kanamycin, capreomycin, amikacin, moxifloxacin, levofloxacin, pyrazinamide according to Cambau et al[1] (critical concentrations listed in Table S6). IML used Sensititer MycoTB plate (TREK Diagnostic Systems, Cleveland, OH) for the determination of MIC of 223 isolates for rifampicin, isoniazid, kanamycin, amikacin, moxifloxacin, levofloxacin, ethambutol (Table S7).

Whole genome sequencing and molecular drug resistance prediction

WGS was performed on full subcultures of clinical isolates using Illumina technology (MiSeq or NextSeq 500, and Nextera XT library preparation kit) according to the manufacturer's instructions. Raw read data (study acc.no PRJEB48275) were mapped to the M. tuberculosis H37Rv genome (GenBank accession no. NC_000962.3) using MTBseq[2] and aimed for at least 50x average genome wide coverage. We considered variants (single nucleotide polymorphisms (SNPs), short insertions and deletions), with a minimum coverage of 10 reads and at least 75% of the reads calling the allele. Here, we report on the predictions of resistance against the following anti-TB drugs and associated resistance genes: rifampicin (rpoB), isoniazid (fabG1, fabG1 promotor, inhA, ndh, katG, mshA, ahpC, ahpC promotor), levofloxacin and moxifloxacin (gyrA, gyrB), kanamicin (rrs, eis promotor) amikacin (rrs), capreomycin (rrs, tlyA), ethambutol, (embCAB operon), pyrazinamide (pncA, pncA promotor, rpsA). In addition, tier 2 genes were verified separately for drug resistant isolates lacking mutations in a tier 1 gene. Genotypic resistance was inferred on the basis of a curated mutation catalogue and global rules, used at the Supranational Reference Laboratory (SRL), Research Center Borstel, Germany, based on information available on 2020-05-10[3]. The catalogue was built on the following rationales as published earlier [3]: 1. inclusion of mutations listed in the

WHO "The use of next-generation sequencing technologies for the detection of mutations associated with drug resistance in Mycobacterium tuberculosis complex: technical guide"[4]; 2. inclusion of the Cryptic mutation catalogue[5] ; 3. inclusion of the interpretation catalogue pncA mutations by considering uncharacterized amino acid changes in pncA as resistance determinant[6] ; 4. individual literature references for the interpretations of mutation implicated in resistance to MDR-TB drugs, 5. information about phylogenetically informative mutations with a likely benign character[7], and 6. unknown insertions and deletions in the following genes were considered as resistance determinants: rpoB (rifampicin, rifabutin), katG (isoniazid), ethA (prothionamide, not relevant in this study), pncA (pyrazinamide), Rv0678c (bedaquiline, clofazimine, not relevant in this study), ald (cycloserine not relevant in this study), tlyA (capreomycin). Heteroresistance and mixed infection detection were not performed systematically in the study. However, for isolates lacking a resistance mediating mutation, employing the above-mentioned threshold for allele frequency and coverage, and exhibiting a MIC above the CC, we applied less strict variant calling thresholds. In table S6 we report all resistance mediating mutations indicated by at least two reads in both forward and reverse orientation.

Data analysis

All data was entered and stored in a database. All analyses were performed using R software[8], and all analyses were descriptive. All variables used in the analysis were taken from the database, no transformations were used and no imputations were performed. Full binary phenotypic resistance profiles of each isolate were inferred from the MIC data based on the previous WHO classification[9]. Not all isolates had MIC results for all 9 antibiotics; for isolates without MIC results, information on antimicrobial resistance and susceptibility was added from available binary pAST results. For levofloxacin, a subset of isolates was tested at the previously endorsed critical concentration[10] (CC) of 1·5 mg/L and MIC dilutions did not include the new CC of 1 mg/L. These isolates were excluded from the sensitivity/specificity analysis as their pAST result was not interpretable Also, some isolates that were tested for INH resistance have reported MIC of ≤0·1, these isolates were not excluded from the analysis, however they were excluded from high-resolution MIC figures as there is not enough resolution for their MIC. Genotypic resistance profile of each isolate was inferred from WGS results. After removal of known phylogenetic mutations and removal of synonymous mutations, each isolate was left with zero, one or more than one mutation in each aforementioned resistance associated gene. gAST (resistant or susceptible) was determined by comparing detected mutations in resistance-associated genes with established list of resistance-causing mutations (Supplemental table S3). If multiple mutations were present in a particular resistance-associated gene and only one of the mutations was a known resistance-causing mutation, then this mutation was considered to be the main resistance-causing

mutation. All non-synonymous mutations detected in resistance-associated genes were always taken together and used in the analysis. To calculate sensitivity and specificity, WGS-based resistance prediction was compared to pAST results using the WHO endorsed CC[10] from 2018, where CC for rifampicin was 1·0 mg/L (Table S6, S7). More specifically, pAST results was considered as a standard, and gAST was compared to the pAST to calculate specificity and sensitivity with the 95% confidence intervals. Co-occurrence of a non-synonymous mutation in resistance-associated gene(s) (that was not part of the established list of resistance-causing mutations) and elevated MIC above the CC for a specific anti-TB drug was considered to be a potential indication for a novel resistance causing mutation. Analysis of MIC variation around the CC in multiple isolates with the same mutation was descriptive. For each mutation (combination of mutations), we analysed the distribution of MIC i.e. whether measured MICs are all a) higher than CC or b) equal or lower to CC. Isolates with particular mutation(s) where MIC are distributed both above the CC and equal/lower than CC, we describe the MIC to vary around the CC, no statistical test was used to describe the MIC variation. Phylogenetic relationships of all analysed Mtbc isolates were inferred from hierarchical clustering based on a distance matrix of 33,605 SNPs[2]. SNP distance matrix was calculated by first aligning all variable positions (SNPs identified in at least one isolate) in all isolates from this study, and then the distance (number of different SNPs) between each pair of isolates was calculated.The resulting dendrogram was visualized using the iTol software[11].After first initial data inspection, two isolates with rpoB S450L mutation and four isolates with katG S315T mutation (one isolate was overlapping, so 5 isolates in total) which had drug susceptible phenotypes for rifampicin and isoniazid, respectively were removed from the analysis as they likely represent labelling error rather than methodical error. Repeated phenotypic testing of these isolates was not performed.

Table S1. Genes analyzed for presence of antibiotic resistance mutations

Antibiotic	Genes (WHO tier 1)	Genes (WHO tier 2)
isoniazid	katG, promoter region fabG1 (inhA), inhA, fabG1, ahpC, promoter region ahpC,	mshA, ndh, Rv2752c, 1258c
rifampicin	rpoB	Rv2752c, rpoA, rpoC
kanamycin	rrs, promoter region eis, whiB7	
amikacin	rrs, promoter region eis	whiB6, ccsA, fprA, aftB
capreomycin	rrs, tlyA	whiB6, ccsA, fprA, aftB
moxifloxacin	gyrA, gyrB	
levofloxacin	gyrA, gyrB	
ethambutol	embA, promoter region embA, embB, embC	ubiA, embR
pyrazinamide	pncA, promoter region pncA, rpsA, panD, clpC1	1258c, PPE35, Rv3236c

*rpsA not part of WHO tier 1 and tier 2 genes

Table S2. All Mtbc strains with corresponding information on mutations, pDST, MIC, lineage, country of origin (Excel)

Table S3. Catalogue of resistance mutations and phylogenetic mutations used for the analysis (Excel)

Table S4. Sensitivity and specificity of WGS-based antibiotic resistance prediction compared to phenotypic DST – MIC performed on MGIT and MycoTB

	MGIT				MycoTB			
Antibiotic	N strains (pDST)		Sensitivity (95% CI)	Specificity (95% CI)	N strains (pDST)		Sensitivity (95% CI)	Specificity (95% CI)
	S	R			S	R		
isoniazid	39	635	98·6 (98·3-98·9)	92·3 (89·4-95·2)	49	173	99·4 (99·0-99·8)	100 (100-100)
rifampicin	57	614	99·3 (99·1-99.6)	36·8 (32·5-41·2)	57	166	100 (100-100)	91·2 (88·7-93·8)
kanamycin	486	186	94·6 (93·5-95·7)	95·3 (94·6-95·9)	78	145	97·9 (97·1-98·7)	93.6 (91·7-95·5)
amikacin	636	36	94·4 (91·9-97·0)	98·4 (98·1-98·8)	78	145	97·9 (97·1-98·7)	100 (100-100)
moxifloxacin	604	70	84·3 (81·4-87·2)	98·8 (98·5-99·1)	77	146	91·1 (89·5-92·7)	84·4 (81·6-87·2)
levofloxacin	613	42	97·6 (96·0-99·2)	98·4 (98·0-98·7)	15	54	92·6 (90·2-95.0)	46·7 (38·0-55·4)

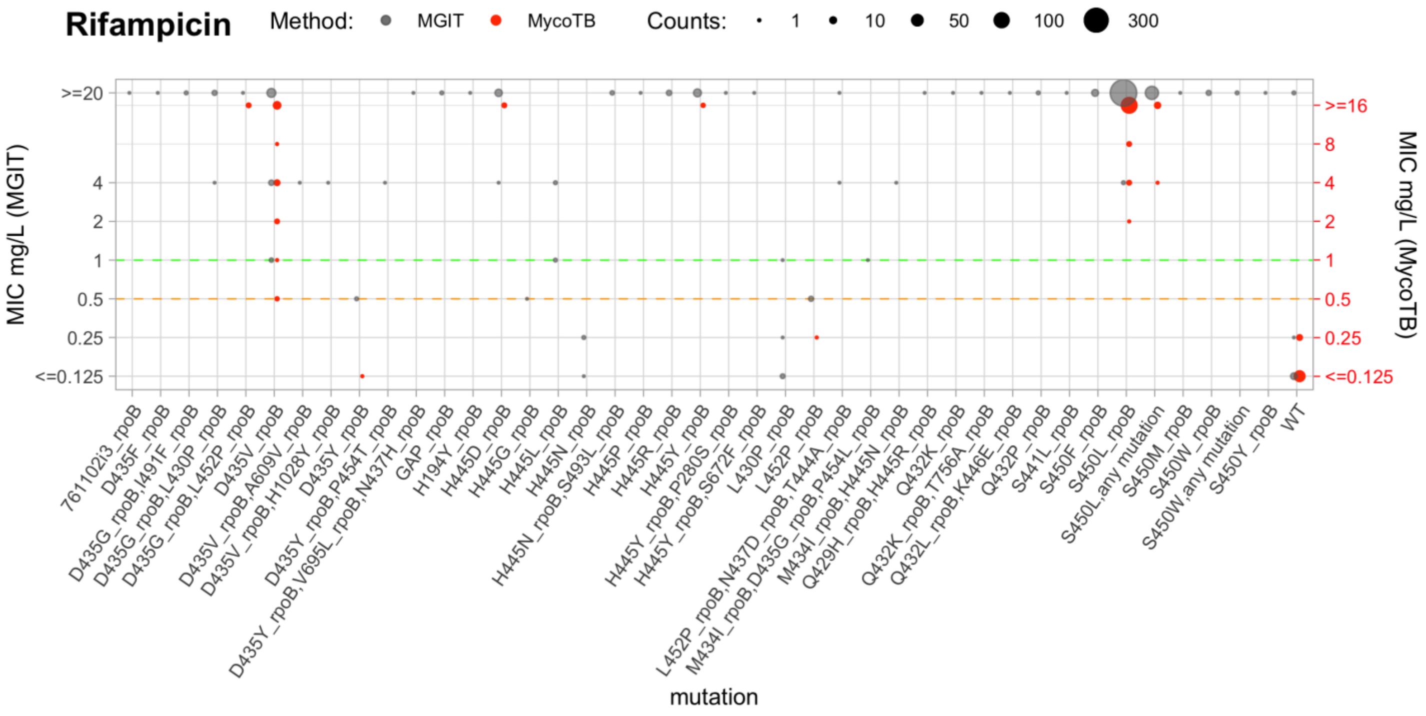

Figure S1. Rifampicin drug resistance mutations with corresponding MIC tested with MGIT and MycoTB

Each circle represents isolates with specific mutation(s) on X-axis and corresponding MIC on y-axis. The size of the circle corresponds to number of isolates with the same mutation(s) and MIC. Grey circles represent strains tested using MGIT, red circles strains tested using MycoTB. Green dashed line represents CC in MGIT. Left y- axis represents all MIC used in MGIT, right y-axis (red) represents MICs used in MycoTB. Strains on and below the green line (CC) are considered susceptible. Analyzed gene: rpoB. “U_” – stands for uncovered, corresponding to large deletions; “GAP_” – stands for deletions. Dashed orange line represents the newest CC for Rifampicin of 0.5 mg/L[12]. 22 isolates in MGIT had MIC <=1.0 and finer differentiation was not available, so these isolates were not included in the figure

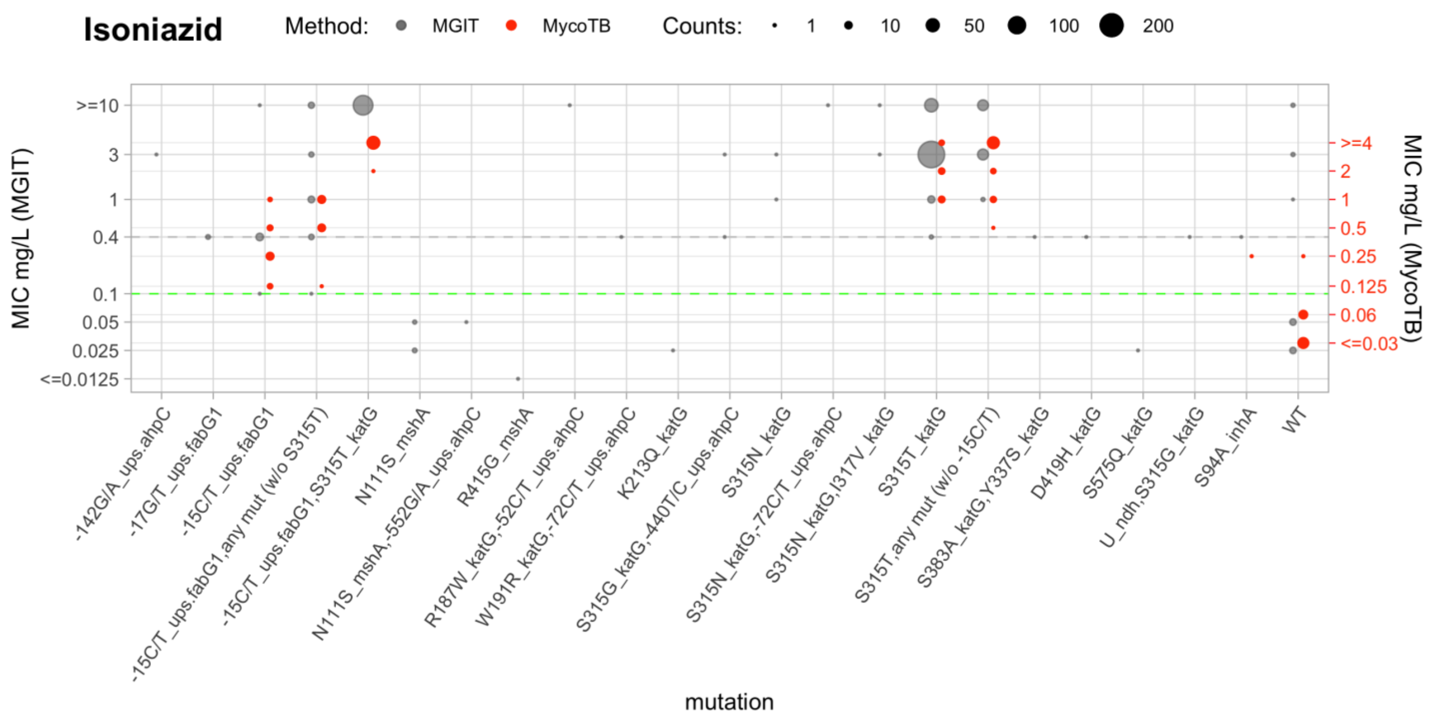

Figure S2. Isoniazid drug resistance mutations with corresponding MIC tested with MGIT and MycoTB

Each circle represents isolates with specific mutation(s) on X-axis and corresponding MIC on y-axis. The size of the circle corresponds to number of isolates with the same mutation(s) and MIC. Grey circles represent strains tested using MGIT, red circles strains tested using MycoTB. Green dashed line represents CC in MGIT. Left y- axis represents all MIC used in MGIT, right y-axis (red) represents MICs used in MycoTB. Strains on and below the green line (CC) are considered susceptible. Analyzed gene: katG, inhA, fabG1, mshA and ndh. “ups” stands for upstream – corresponding to promoter region. “S315T_katG, any mut (w/o – 15C/T_ups.gabG1)” – corresponds to strains that in addition to mutation S315T in katG gene have other mutations in analysed genes excluding -15 C/T in promoter region fabG1; “-15C/T_ups.fabG1, any mut (w/o S315T_KatG) – corresponds to strains that in addition to mutation -15 C/T in promoter region of fabG1 gene have other mutations in analysed genes excluding S315T in katG, “U_” – stands for uncovered, corresponding to large deletions; “GAP_” – stands for deletions. Grey dashed line represents CC of 0.4 mg/L depicting threshold between low (0.1 mg/L < MIC ≤ 0.4 mg/L) and high-level resistant isolates (MIC >0.4 mg/L). 12 isolates in MGIT had MIC <=0.1 and finer differentiation was not available, so these isolates were not included in the figure.

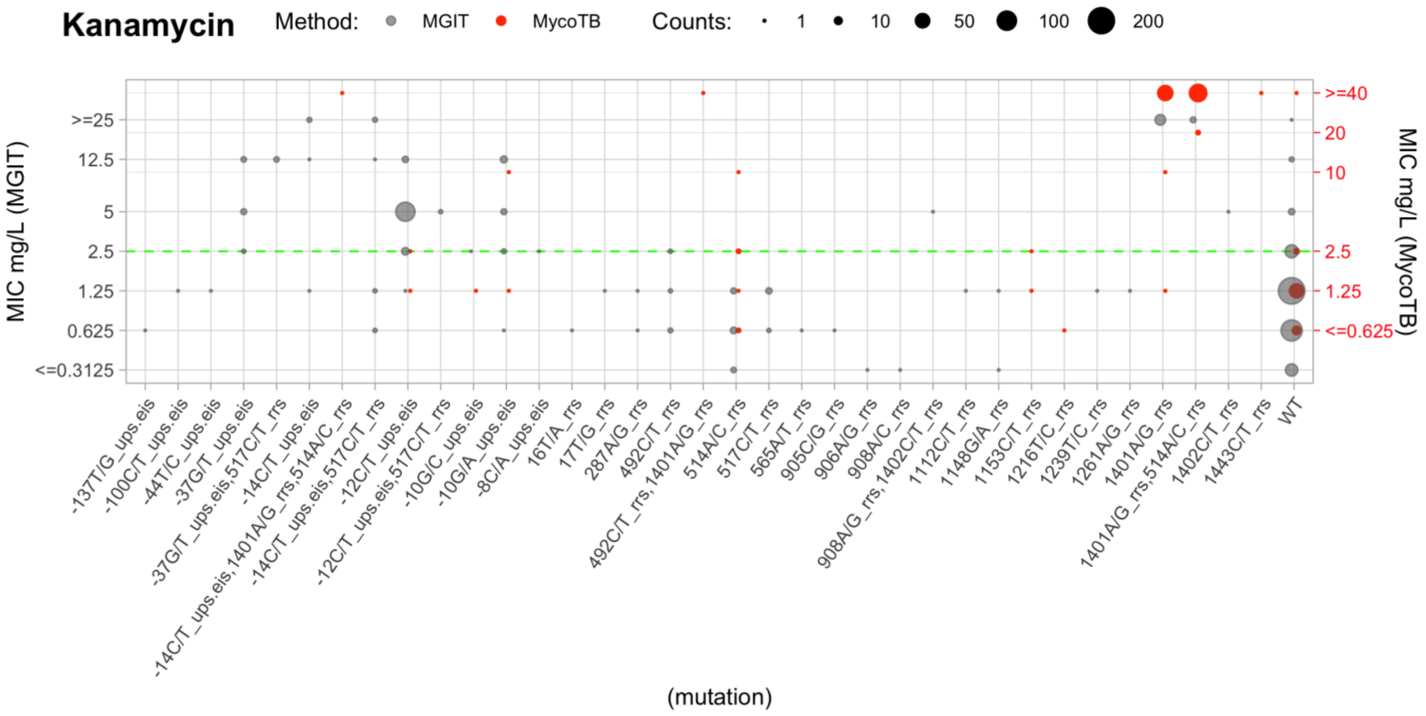

Figure S3. Kanamycin drug resistance mutations with corresponding MIC tested with MGIT and MycoTB

Each circle represents isolates with specific mutation(s) on X-axis and corresponding MIC on y-axis. The size of the circle corresponds to number of isolates with the same mutation(s) and MIC. Grey circles represent strains tested using MGIT, red circles strains tested using MycoTB. Green dashed line represents CC in MGIT. Left y- axis represents all MIC used in MGIT, right y-axis (red) represents MICs used in MycoTB. Strains on and below the green line (CC) are considered susceptible. Analyzed gene: rrs and eis. “ups” stands for upstream – corresponding to promoter region.

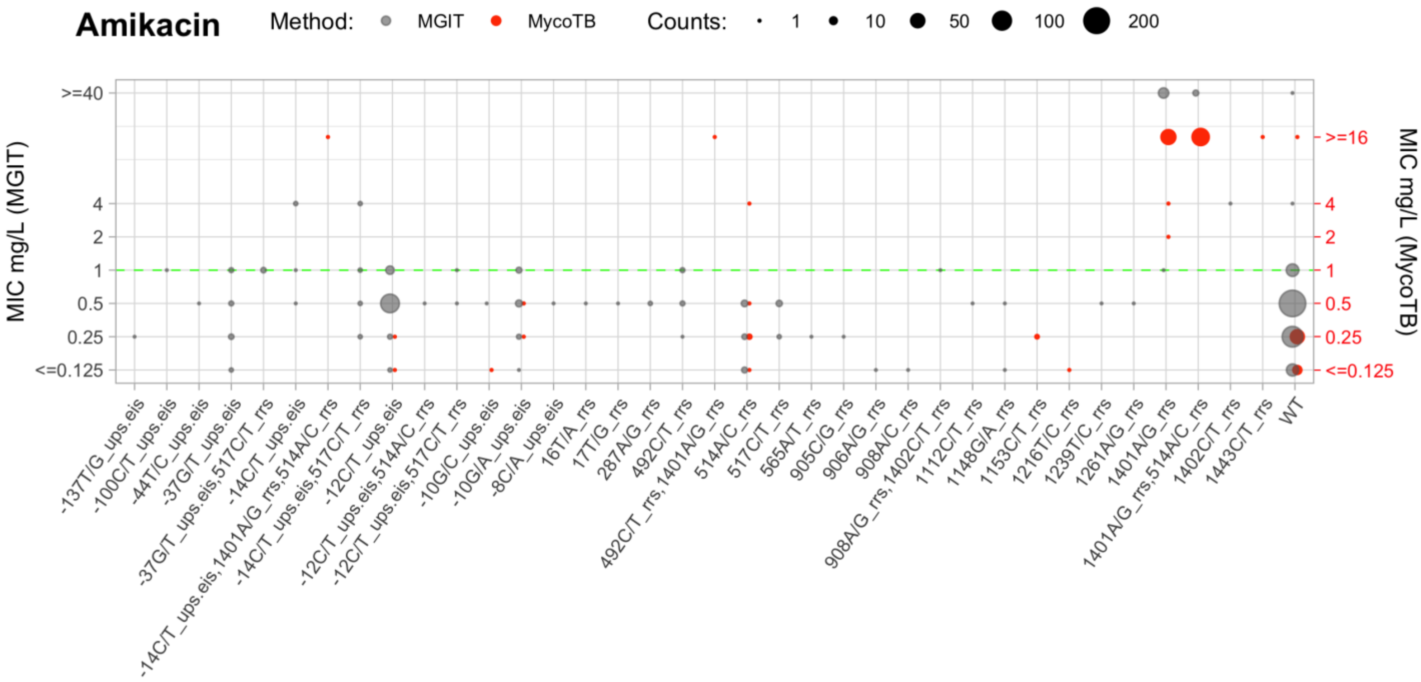

Figure S4. Amikacin drug resistance mutations with corresponding MIC tested with MGIT and MycoTB
Each circle represents isolates with specific mutation(s) on X-axis and corresponding MIC on y-axis. The size of the circle corresponds to number of isolates with the same mutation(s) and MIC. Grey circles represent strains tested using MGIT, red circles strains tested using MycoTB. Green dashed line represents CC in MGIT. Left y- axis represents all MIC used in MGIT, right y-axis (red) represents MICs used in MycoTB. Strains on and below the green line (CC) are considered susceptible. Analyzed gene: rrs and eis. “ups” stands for upstream – corresponding to promoter region.

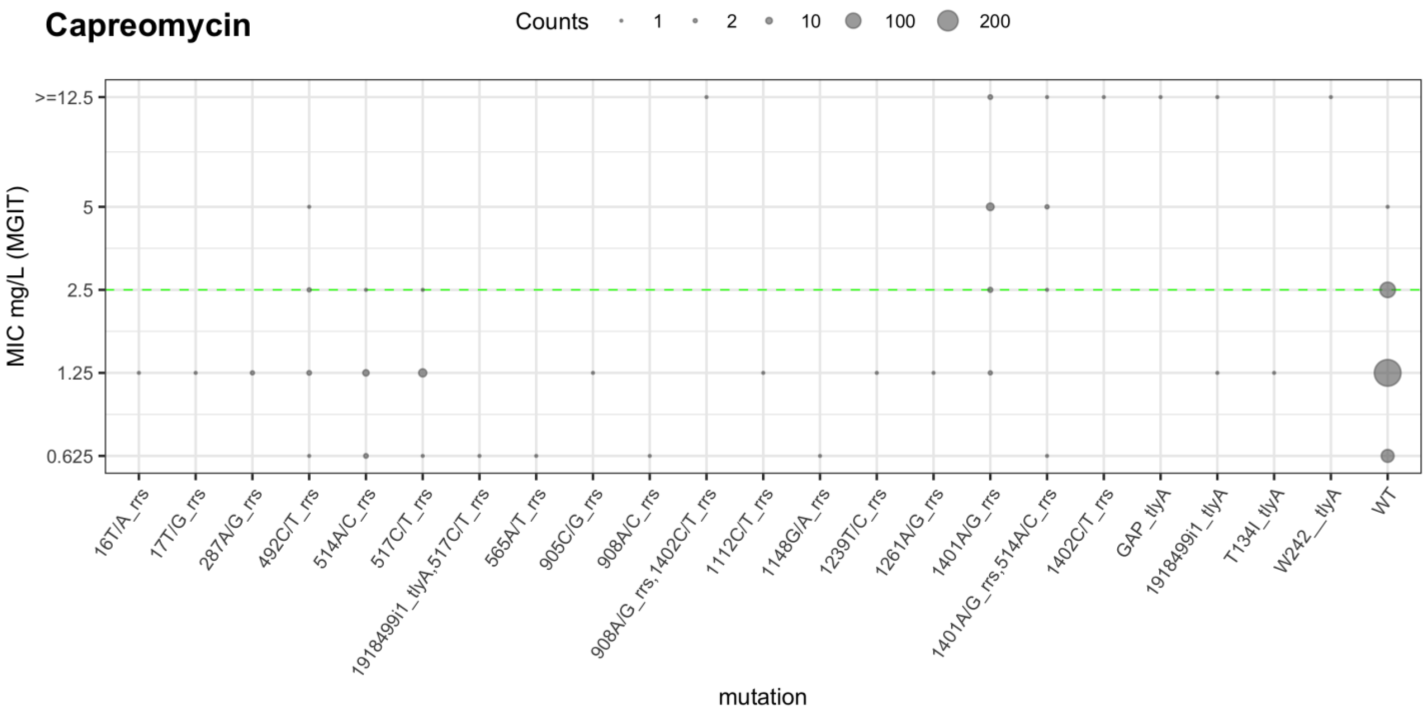

Figure S5. Capreomycin drug resistance mutations with corresponding MIC tested with MGIT

Each circle represents isolates with specific mutation(s) on X-axis and corresponding MIC on y-axis. The size of the circle corresponds to number of isolates with the same mutation(s) and MIC. Green dashed line represents CC in MGIT. Strains on and below the green line (CC) are considered susceptible. Analyzed gene: rrs and tlyA.

“GAP_” – stands for deletions

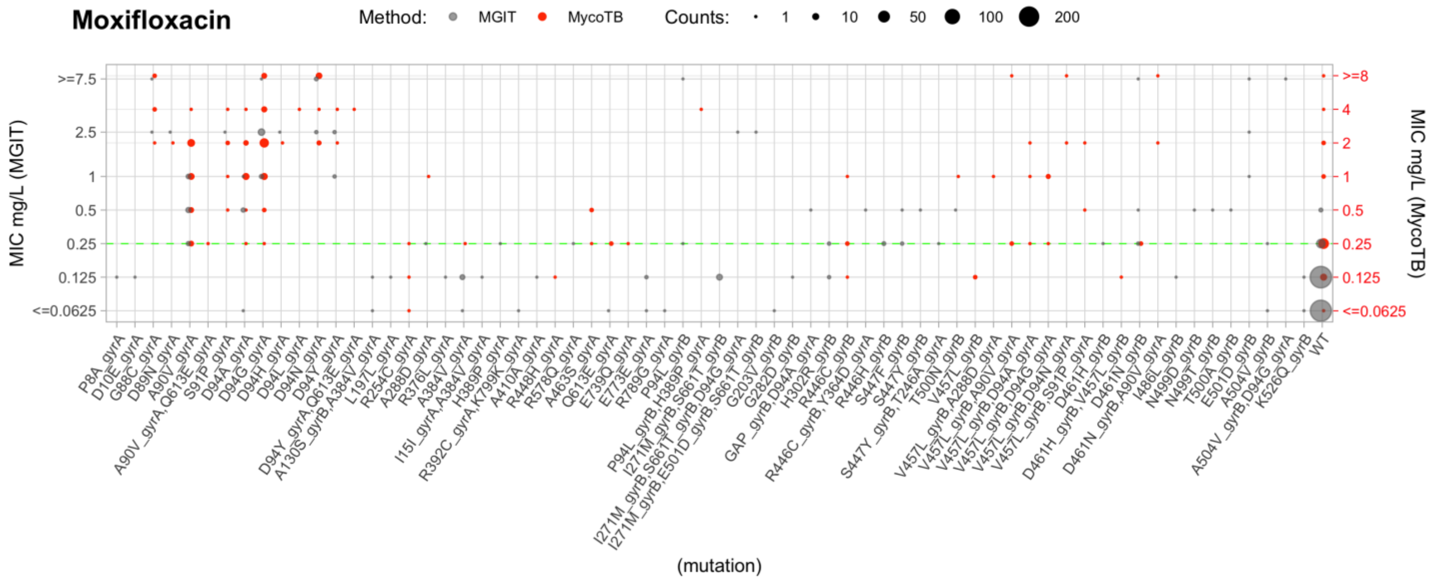

Figure S6. Moxifloxacin drug resistance mutations with corresponding MIC tested with MGIT and MycoTB
Each circle represents isolates with specific mutation(s) on X-axis and corresponding MIC on y-axis. The size of the circle corresponds to number of isolates with the same mutation(s) and MIC. Grey circles represent strains tested using MGIT, red circles strains tested using MycoTB. Green dashed line represents CC in MGIT. Left y- axis represents all MIC used in MGIT, right y-axis (red) represents MICs used in MycoTB. Strains on and below the green line (CC) are considered susceptible. Analyzed gene: gyrA and gyrB. GAP_" – stands for deletions

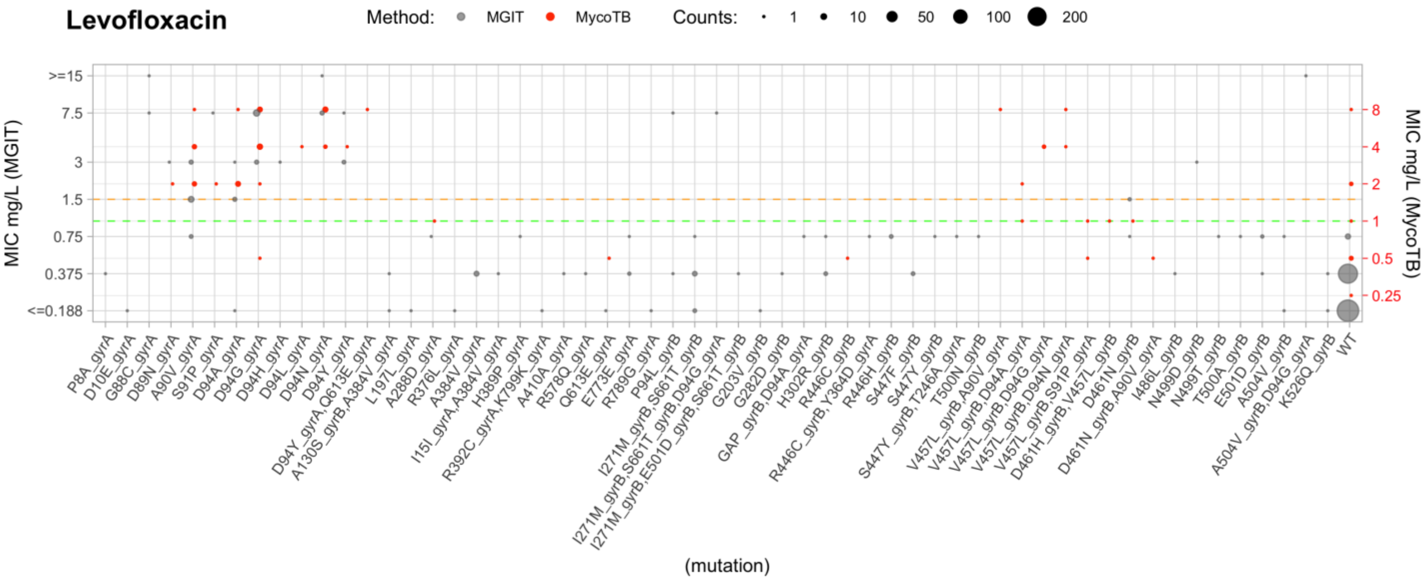

Figure S7. Levofloxacin drug resistance mutations with corresponding MIC tested with MGIT and MycoTB

Each circle represents isolates with specific mutation(s) on X-axis and corresponding MIC on y-axis. The size of the circle corresponds to number of isolates with the same mutation(s) and MIC. Grey circles represent strains tested using MGIT, red circles strains tested using MycoTB. Green dashed line represents CC in MGIT. Left y- axis represents all MIC used in MGIT, right y-axis (red) represents MICs used in MycoTB. Strains on and below the green line (CC) are considered susceptible. Orange dashed line represents old CC, at which some samples were tested. Analyzed gene: gyrA and gyrB. GAP_" – stands for deletions

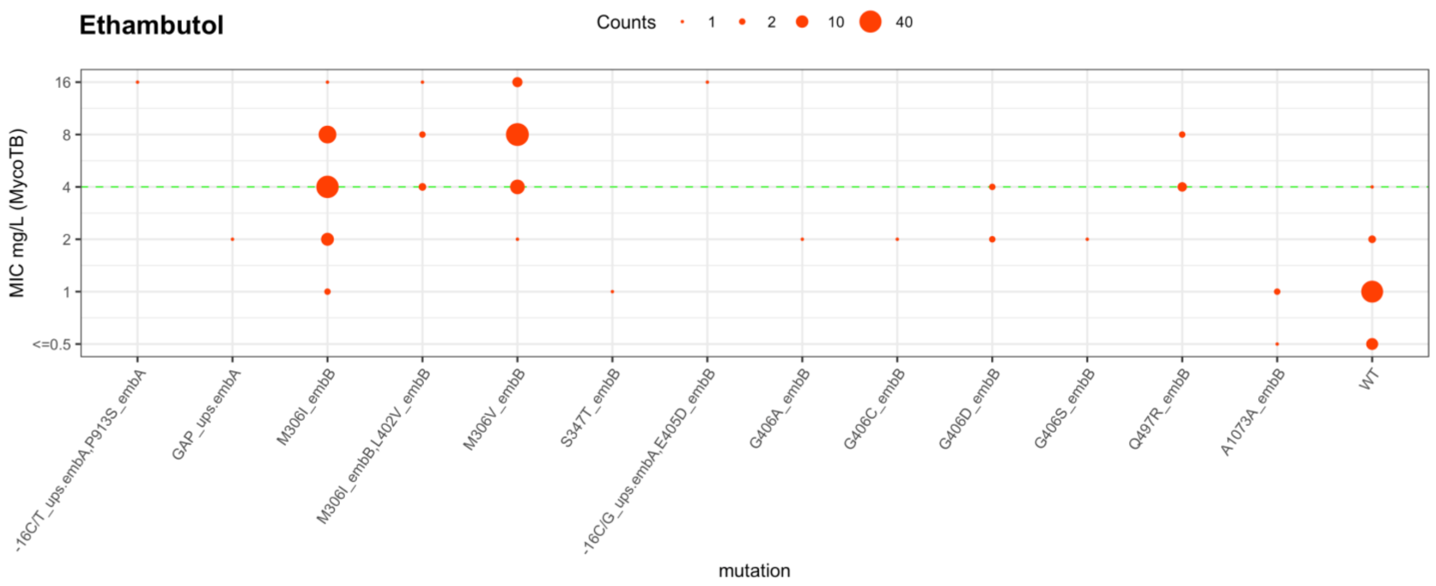

Figure S8. Ethambutol drug resistance mutations with corresponding MIC tested with MycoTB

Each circle represents isolates with specific mutation(s) on X-axis and corresponding MIC on y-axis. The size of the circle corresponds to number of isolates with the same mutation(s) and MIC. Green dashed line represents CC in MGIT. Strains on and below the green line (CC) are considered susceptible. Analyzed gene: embA, embB and embC. “ups” stands for upstream – corresponding to promoter region. GAP_” – stands for deletions

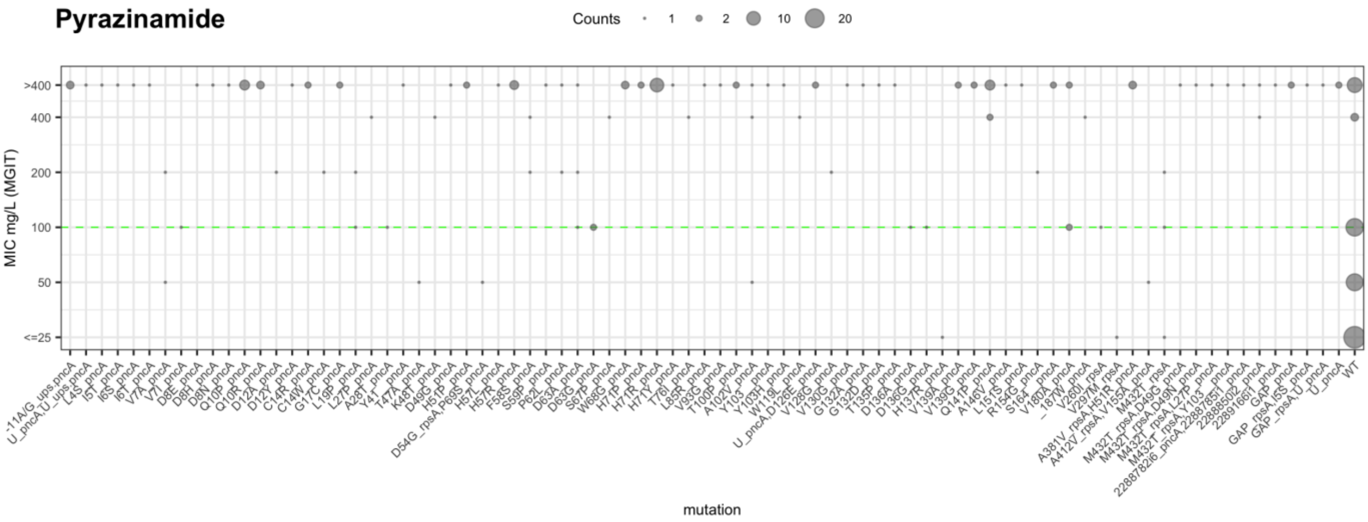

Figure S9. Pyrazinamide drug resistance mutations with corresponding MIC tested with MGIT

Each circle represents isolates with specific mutation(s) on X-axis and corresponding MIC on y-axis. The size of the circle corresponds to number of isolates with the same mutation(s) and MIC. Green dashed line represents CC in MGIT. Strains on and below the green line (CC) are considered susceptible. Analyzed gene: pncA and rpsA. “ups” stands for upstream – corresponding to promoter region. “U_” – stands for uncovered, corresponding to large deletions; GAP_” – stands for deletions

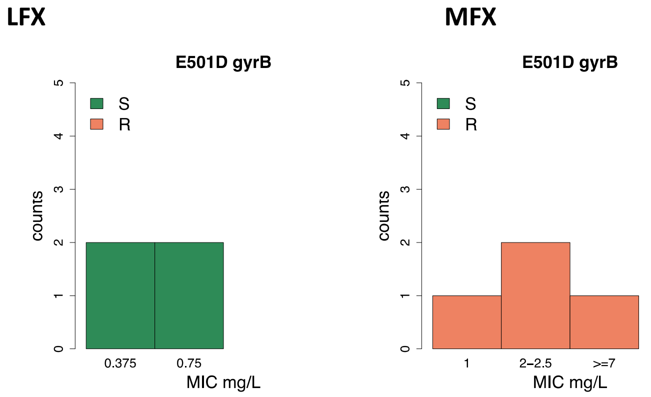

Figure S10. Mutation E501D in gyrB causing different MIC in moxifloxacin and levofloxacin

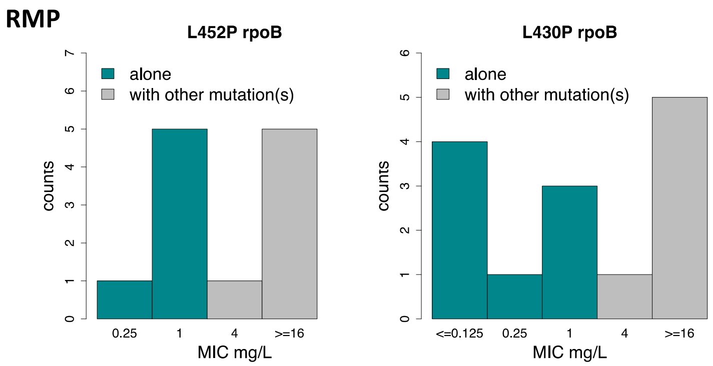

Figure S11. MIC distribution for strains with mutations L452P and L430P in rpoB alone or with other mutations in rpoB

Table S5. MIC close to the critical concentration

Antibiotic	Gene	Mutation	N all isolates	N resistant isolates
Moxifloxacin	gyrA	A288D	2	1
	gyrB	P94L	2	1
	gyrB	R446C	4	1
	gyrB	S447F	3	1
	gyrA	A463S	3	2
	gyrB	D461N	6	2
Levofloxacin	gyrB	P94L	2	1
Pyrazinamide	pncA	A102V	3	2
	rpsA	M432T	3	1

N – number

Table S6. Examination of low frequency or low coverage mutations in strains with antibiotic resistance predicted based on pDST where gDST characterization predicted WT strains

antibiotic	N	gDST characterization (>75% mutation frequency and >10 reads coverage)	low frequency/low coverage mode (at least 2 reads in both forward and reverse orientation showing the same allele)	frequency	MIC (mg/L)
rifampicin	1	WT	rpoB S450L	5.3%	20
isoniazid	1	WT	katG S315T	10.7 %	3
isoniazid	1	WT	fabG1 -15c>t	16.5%	0.25
kanamycin, amikacin	1	WT	rrs 1401a>g	42.48%	>25 (KAN); >40 (AMK)

kanamycin, amikacin	1	WT	rrs 1401a>g	32.47%	40 (KAN); 16 (AMK)
moxifloxacin, levofloxacin	1	WT	gyrA A90V; gyrA S91P	48.32%; 40 %	2 (MFX); 2 (LFX)
moxifloxacin, levofloxacin	1	WT	gyrB N499D	6.6 %	2 (MFX); 2 (LFX)
moxifloxacin	1	WT	gyrA D94G	45.59%	4
moxifloxacin, levofloxacin		WT	gyrA A90V; gyrA D94N	36.57%; 11.35%	8 (MFX); 8 (LFX)
pyrazinamide	1	WT	pncA A171E	18.03%	400
pyrazinamide	2	WT; WT	pncA 259_ins_caggtctgaccctaccttcaggcgc	43.75%; 47.97 %	>400; 400
pyrazinamide	1	WT	pncA Y99_; pncA 193_ins_a	21.61%; 71.34%	>400
pyrazinamide	1	WT	pncA 122_del_a	34.56%	>400
pyrazinamide	1	WT	pncA C72Y	100%	>400
pyrazinamide	1	WT	pncA 394_ins_c	98.45%	>400
pyrazinamide	1	WT	pncA 450_ins_g	94.44%	>400
pyrazinamide	1	WT	pncA T142M	100%	>400
pyrazinamide	1	WT	pncA Q122_	100%	>400

Table S7. Concentrations used for testing performed in BD MGIT 960

Drug	CC[10] (mg/l)	Susceptible			Resistant			
		c1	c2	c3	c1	c2	c3	c4
Rifampicin	1	0·5	0. ·5	0·125	4	20		
Isoniazid	0·1	0·05	0·025	0·0125	0·4	1	3	10
Levofloxacin	1·5	0·75	0·375	0·1875	3	7·5	15	
Moxifloxacin	0·5	0·25	0·125	0·0625	1	2·5	7·5	
Kanamycin	2·5	1·25	0·625	0·3125	5	12·5	25	
Amikacin	1	0·5	0·25	0·125	4	20	40	
Capreomycin	2·5	1·25	0·625	0·3125	5	12·5	25	
Pyrazinamide	100	50	25		200	400		

c - concentration (mg/l), CC – critical concentration

Table S8. Concentrations used for testing performed in Sensititre MycoTB plate

Drug	CC[10] (mg/l)	Susceptible			Resistant				
		c1	c2	c3	c1	c2	c3	c4	c5
Rifampicin	1	0·5	0·25	0·12	2	4	8	16	
Isoniazid	0·1	0·125	0·06	0·03	0·25	0·5	1	2	4
Levofloxacin	1	0·5	0·25	0·12	2	4	8		
Moxifloxacin	0·5	0·25	0·12	0·06	1	2	4	8	
Kanamycin	2·5	1·25	0·625	0·3125	5	10	20	40	
Amikacin	1	0·5	0·25	0·12	2	4	16		
Ethambutol	4	2	1	0·5	8	16			

c - concentration (mg/l), CC – critical concentration

References

1 Cambau E, Viveiros M, Machado D, *et al.* Revisiting susceptibility testing in MDR-TB by a standardized quantitative phenotypic assessment in a European multicentre study. J Antimicrob Chemother 2015; 70: 686–96.

2 Kohl TA, Utpatel C, Schleusener V, et al. MTBseq: a comprehensive pipeline for whole genome sequence analysis of Mycobacterium tuberculosis complex isolates. PeerJ 2018; 6: e5895.

3 Grobbel H-P, Merker M, Köhler N, et al. Design of Multidrug-Resistant Tuberculosis Treatment Regimens Based on DNA Sequencing. Clin Infect Dis 2021; 73: 1194–202.

4 WHO. The use of next-generation sequencing technologies for the detection of mutations associated with drug resistance in Mycobacterium tuberculosis complex: technical guide. 2018 DOI:10.7868/s0023476118010137.

5 The CRyPTIC Consortium and the 100 000 Genomes Project. Prediction of Susceptibility to First-Line Tuberculosis Drugs by DNA Sequencing. NEJM 2018; 379: 1403–15.

6 Yadon AN, Maharaj K, Adamson JH, et al. A comprehensive characterization of PncA polymorphisms that confer resistance to pyrazinamide. Nat Commun 2017; 8: 588.

7 Merker M, Kohl TA, Barilar I, et al. Phylogenetically informative mutations in genes implicated in antibiotic resistance in Mycobacterium tuberculosis complex. Genome Med 2020; 12: 27.

8 R Foundation for Statistical Computing. R Core Team (2018). R: A language and environment for statistical computing. https://www.r-project.org/.

9 WHO. TB drug resistance types. https://www.who.int/tb/areas-of-work/drug-resistant-tb/types/en/.

10 WHO. Technical manual for drug susceptibility testing of medicines used in the treatment of tuberculosis. 2018.

11 Letunic I, Bork P. Interactive Tree Of Life (iTOL) v4 : recent updates and new developments. Nucleic Acids Res 2019; 47: 256–9.

12 World Health Organisation. Technical Report on critical concentrations for drug susceptibility testing of isoniazid and the rifamycins (rifampicin, rifabutin and rifapentine). 2021.
